# 4-[3-(1*H*-Imidazol-1-yl)prop­yl]-3-methyl-5-(thio­phen-2-ylmeth­yl)-4*H*-1,2,4-triazole monohydrate

**DOI:** 10.1107/S160053681004571X

**Published:** 2010-11-13

**Authors:** Anuradha Gurumoorthy, Vasuki Gopalsamy, Dilek Ünlüer, Gülcan Kör, K. Ramamurthi

**Affiliations:** a Department of Physics, Saveetha School of Engineering, Saveetha University, Chennai-5, India; b Department of Physics, Kunthavai Naachiar Government Arts College (w) (Autonomous), Thanjavur-7, India; c Department of Chemistry, Faculty of Arts and Sciences, Karadeniz Teknik University, Trabzon 61080, Turkey; d Crystal Growth and Thin Film Laboratory, School of Physics, Bharathidasan University, Tiruchirappalli-24, India

## Abstract

In the title compound, C_14_H_17_N_5_S·H_2_O, the triazole ring makes dihedral angles of 48.15 (8) and 84.92 (8)° with the imidazole and thio­phenyl rings, respectively. The water mol­ecule is involved in inter­molecular O—H⋯N hydrogen bonding.

## Related literature

For details of the synthesis, see: Ünver *et al.* (2009 ▶). For related structures, see: Fun *et al.* (2010 ▶); Kalkan *et al.* (2007 ▶); Ustabaş *et al.* (2007 ▶, 2009 ▶); Ünver *et al.* (2006 ▶). For the biological activity of triazoles, see: Ustabaş, *et al.* (2006*a* ▶,*b* ▶); Yılmaz *et al.* (2006 ▶). For bond-length data, see: Allen *et al.* (1987 ▶). 
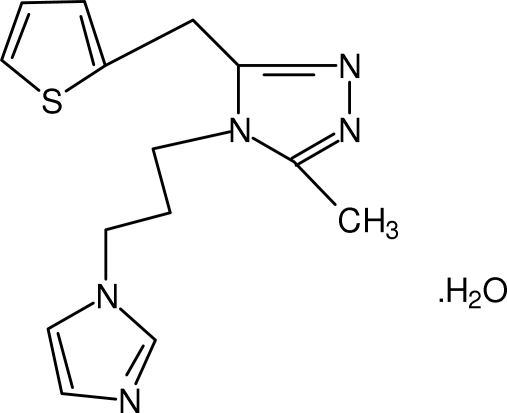


## Experimental

### 

#### Crystal data

C_14_H_17_N_5_S·H_2_O
*M* *_r_* = 305.40
Monoclinic, 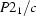

*a* = 9.5584 (12) Å
*b* = 9.4873 (10) Å
*c* = 17.644 (3) Å
β = 99.360 (12)°
*V* = 1578.7 (4) Å^3^
*Z* = 4
Cu *K*α radiation
μ = 1.88 mm^−1^
*T* = 293 K
0.30 × 0.25 × 0.20 mm

#### Data collection

Enraf–Nonius CAD-4 diffractometer
Absorption correction: ψ scan (North *et al.*, 1968 ▶) *T*_min_ = 0.603, *T*_max_ = 0.705
2855 measured reflections
2679 independent reflections
2336 reflections with *I* > 2σ(*I*)
*R*_int_ = 0.016

#### Refinement

*R*[*F* ^2^ > 2σ(*F* ^2^)] = 0.051
*wR*(*F* ^2^) = 0.137
*S* = 1.12
2679 reflections
200 parameters
3 restraints
H atoms treated by a mixture of independent and constrained refinement
Δρ_max_ = 0.31 e Å^−3^
Δρ_min_ = −0.34 e Å^−3^

### 

Data collection: *CAD-4 EXPRESS* (Enraf–Nonius, 1994 ▶); cell refinement: *CAD-4 EXPRESS*; data reduction: *MolEN* (Fair, 1990 ▶); program(s) used to solve structure: *SHELXS97* (Sheldrick, 2008 ▶); program(s) used to refine structure: *SHELXL97* (Sheldrick, 2008 ▶); molecular graphics: *PLATON* (Spek, 2009 ▶) and *ZORTEP* (Zsolnai, 1997 ▶); software used to prepare material for publication: *SHELXL97*.

## Supplementary Material

Crystal structure: contains datablocks I, global. DOI: 10.1107/S160053681004571X/jh2226sup1.cif

Structure factors: contains datablocks I. DOI: 10.1107/S160053681004571X/jh2226Isup2.hkl

Additional supplementary materials:  crystallographic information; 3D view; checkCIF report

## Figures and Tables

**Table 1:** Hydrogen-bond geometry (Å, °)

*D*—H⋯*A*	*D*—H	H⋯*A*	*D*⋯*A*	*D*—H⋯*A*
O1*W*—H2*W*⋯N5^i^	0.93 (1)	2.04 (2)	2.915 (4)	155 (4)
O1*W*—H1*W*⋯N2	0.93 (1)	2.05 (2)	2.948 (3)	161 (4)

Symmetry code: (i) 
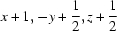
.

**Fig. 1.**
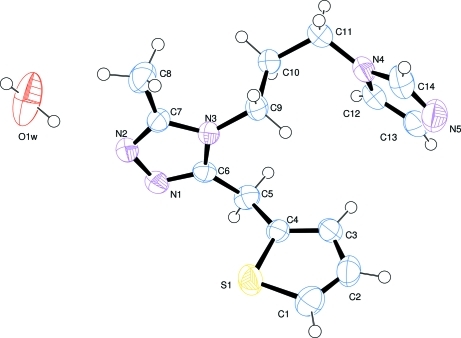
The molecular structure of the title compound. Displacement ellipsoids are drawn at the 50% probability level.

**Fig. 2.**
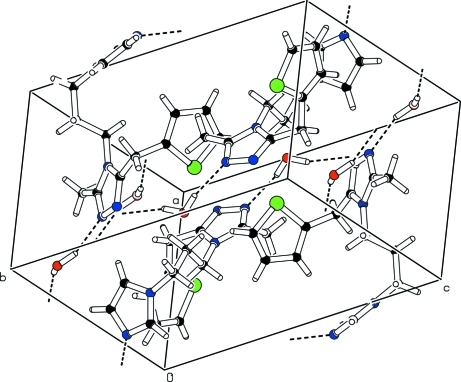
Crystal packing of the title compound viewed along the c axis. Intermolecular O—H···N hydrogen bonds are shown as dashed lines.

**Fig. 3.**
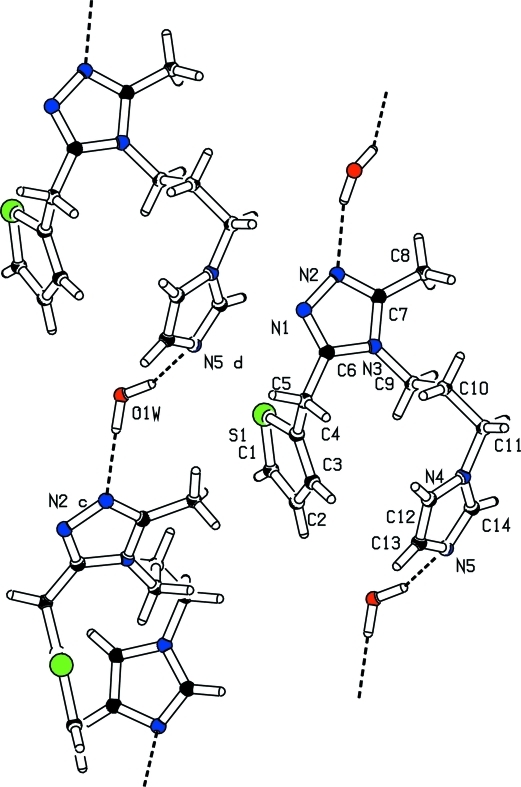
Crystal Packing of the title compound with hydrogen bonds.

**Fig. 4.**
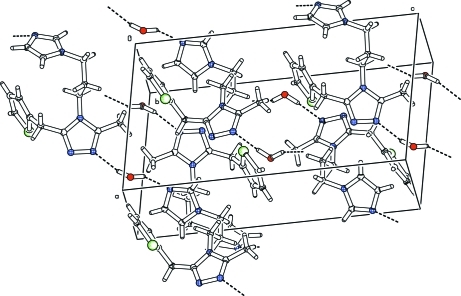
Crystal packing of the title compound viewed along the c axis. Intermolecular O—H···N hydrogen bonds are shown as dashed lines.

## supplementary crystallographic information

### Comment

Triazole compounds and their derivatives have many applications in industry and medicine. Ionic liquids consisting of imidazolium and triazolium salts have attracted increasing interest as an alternative to classical organic solvents for a wide range of chemical syntheses, biocatalysis, electrochemical applications, energetic materials, nano-rods, liquid-liquid separation and polymerization. These interesting liquids containing imidazole and triazole ring systems have unique physical and chemical properties: low melting point, very low vapour pressure, a large liquid phase range, tunable miscibility, and good hydrolytic and thermal stability (Ustabaş *et al.*, 2006). In the field of medicine triazole derivatives were reported to exhibit various pharmacological activities such as antimicrobial, analgesic, anti- inflammatory, anticancer and antioxidant properties. A few derivatives of triazoles have exhibited antimicrobial activity. Some of the drugs such as ribavirin (antiviral agent), rizatriptan (anti migraine agent), alprazolam (anxiolytic agent), fluconazole and itraconazole (antifungal agents) are the best examples for potent molecules possessing the triazole nucleus (Fun *et al.*, 2010). Furthermore, in many compounds, the thiophene unit is associated with high anticancer and antifungal activity (Kalkan *et al.*, 2007). In a previous paper, we reported the 1,2,4 triazole derivative with different substituents. We report here the crystal structure of the title compound (I) (Fig.1) in order to examine the structure activity of 1,2,4 triazole with a thiophene substituent.

Compound (I) contains three planar rings (Fig.1), namely a triazole ring N1/N2/C7/N3/C6 (A), an imidazole ring N4/C12/C13/N5/C14 (B) and a thiophene ring C1/C2/C3/C4/S1 (C). The dihedral angle between rings A/B, A/C and B/C are 48.15 (8)°, 84.92 (8)° and 74.73 (9)°, respectively. In the molecule of the title compound (Fig. 1), the bond lengths (Allen *et al.*, 1987) and angles are within normal ranges. The C—N bond lengths in the triazole ring of all molecules lie in the range of 1.260 (3)–1.349 (4) Å. These are longer than a typical double C═N bond [1.269 (2) Å], but shorter than a C—N single bond [1.443 (4) Å]. The bond length N2—C7 [1.306 (3) Å] and N3—C7 [1.365 (3) Å] are in agreement with the corresponding values in similar structures containing triazole ring such as [1.290 (3)Å and 1.384 (3) Å; Yılmaz *et al.*, 2006], [1.287 (4)Å and 1.374 (4) Å; Ustabaş *et al.*, 2007] and [1.292 (3)Å and 1.373 (3) Å; Ustabaş *et al.*, 2009]. The N1—N2 [1.389 (3) Å] bond length is close to that reported for similar compounds [1.388 (2)Å Ünver *et al.*, 2006] and [1.398 (4)Å Ustabaş, Çoruh, Sancak, Ünver & Vázquez-López (2006)]. Atom N3 has a trigonal configuration, the sum of three bond angles around them being 360° (Kalkan *et al.*, 2007). The bond lengths and angles in the imidazole and thiophenyl rings are normal. In the thiophenyl ring S1—C4 [1.711 (2) Å] bond is longer than S1—C1 [1.699 (3) Å] bond. These S—C distances are in agreement with the corresponding values of those found in other structures containing thiophene, [1.706 (2)Å and 1.723 (2) Å; Ünver *et al.*, 2006], [1.710 (5)Å and 1.724 (4) Å; Ustabaş,Çoruh, Sancak, Ünver & Vázquez-López (2006)],[1.701 (3)Å and 1.712 (2) Å; Yılmaz *et al.*, 2006], [1.685 (5)Å and 1.692 (6) Å; Ustabaş *et al.*, 2007] and [1.698 (3)Å and 1.735 (6) Å; Ustabaş *et al.*, 2009]. The exocyclic bond angles N1—C6—C5 and N2—C7—C8 are 125.08 (18)° and 125.73 (19)°, respectively. These increase from the normal value of 120° might be the consequence of repulsion between the lone pair of electrons on atom N1 and H5B attached to C5(N1···H5B = 2.563 Å) and on atom N2 and H8A attached to C8(N2···H8B = 2.566 Å), respectively. The widening of the excocyclic angles N3—C9—C10 [112.3 (16)°]and C9—C10—C11 [112.5 (19)°] from the normal 109° may be due to the steric repulsion between H8B and H10A (H8B ··· H10A = 2.547 Å) and H9A and H10B (H9A··· H10B = 2.357 Å), respectively. The C10—C11—N4 exocyclic angle [112.13 (18)°] deviate from the normal value of 109° may be due to the consequence of repulsion between the lone pair of electrons on atom N4 and H11A and H11B attached to C11 (N4···H11A = 1.998Å and N4···H11B = 1.998 Å). The C11—N4—C14 exocyclic angle [127.4 (2)°] significantly deviate from the normal value of 120° may be due to the consequence of repulsion between the lone pair of electrons on atom N4 and H14 at C14 (N4···H14 = 2.019 Å). The N3—C9—C10—C11 torsion angle of 178.95 (18)° indicates that the triazole ring and the immidazole moiety has an E-Configuration across the C9—C10 bond. The C9—N3—C6—C5 torsion angle of 4.3 (3)° indicates that the imidazole moiety and the thiophenyl moiety are in *Z*-Configuration across the N3—N6 bond. The water molecule is involved in the intermolecular O–H···N hydrogen bonding (Table 2), which is effective in stabilizing the crystal structure.

### Experimental

The compound was synthesized by published method (Ünver *et al.*,2009)

### Refinement

Water H atoms were located in a difference Fourier map and isotropically refined with O—H distance restraints of 0.90 (1) Å. All the other H atoms were positioned geometrically and treated as riding on their parent atoms, with C—H = 0.93(aromatic),0.96(methyl) and 0.97Å (methylene),*N*—H = 0.86Å and refined using a riding model with *U*_iso_(H) = 1.2Ueq or 1.5Ueq (parent atom).In the absence of significant anomalous scattering effects,Friedel pairs were merged.

### Crystal data

**Table:**

C_14_H_17_N_5_S·H_2_O	*F*(000) = 648
*M**_r_* = 305.40	*D*_x_ = 1.285 Mg m^−^^3^
Monoclinic, *P*2_1_/*c*	Cu *K*α radiation, λ = 1.54180 Å
*a* = 9.5584 (12) Å	Cell parameters from 25 reflections
*b* = 9.4873 (10) Å	θ = 20–30°
*c* = 17.644 (3) Å	µ = 1.88 mm^−^^1^
β = 99.360 (12)°	*T* = 293 K
*V* = 1578.7 (4) Å^3^	Block, colourless
*Z* = 4	0.30 × 0.25 × 0.20 mm

### Data collection

**Table:**

Enraf–Nonius CAD-4 diffractometer	2679 independent reflections
Radiation source: fine-focus sealed tube	2336 reflections with *I* > 2σ(*I*)
graphite	*R*_int_ = 0.016
Detector resolution: 2 pixels mm^-1^	θ_max_ = 64.9°, θ_min_ = 4.7°
ω–2θ scans	*h* = 0→11
Absorption correction: ψ scan (North *et al.*, 1968)	*k* = 0→11
*T*_min_ = 0.603, *T*_max_ = 0.705	*l* = −20→20
2855 measured reflections	

### Refinement

**Table:**

Refinement on *F*^2^	Secondary atom site location: difference Fourier map
Least-squares matrix: full	Hydrogen site location: inferred from neighbouring sites
*R*[*F*^2^ > 2σ(*F*^2^)] = 0.051	H atoms treated by a mixture of independent and constrained refinement
*wR*(*F*^2^) = 0.137	*w* = 1/[σ^2^(*F*_o_^2^) + (0.0774*P*)^2^ + 0.5509*P*] where *P* = (*F*_o_^2^ + 2*F*_c_^2^)/3
*S* = 1.12	(Δ/σ)_max_ = 0.001
2679 reflections	Δρ_max_ = 0.31 e Å^−^^3^
200 parameters	Δρ_min_ = −0.34 e Å^−^^3^
3 restraints	Extinction correction: *SHELXL97* (Sheldrick, 2008), Fc^*^=kFc[1+0.001xFc^2^λ^3^/sin(2θ)]^-1/4^
Primary atom site location: structure-invariant direct methods	Extinction coefficient: 0.042 (2)

### Special details

**Table:**

Experimental. North A.C.T., Phillips D.C. & Mathews F.S. (1968) Acta. Cryst. A24, 351 Number of psi-scan sets used was 5 Theta correction was applied. Averaged transmission function was used. No Fourier smoothing was applied.
Geometry. All e.s.d.'s (except the e.s.d. in the dihedral angle between two l.s. planes) are estimated using the full covariance matrix. The cell e.s.d.'s are taken into account individually in the estimation of e.s.d.'s in distances, angles and torsion angles; correlations between e.s.d.'s in cell parameters are only used when they are defined by crystal symmetry. An approximate (isotropic) treatment of cell e.s.d.'s is used for estimating e.s.d.'s involving l.s. planes.
Refinement. Refinement of *F*^2^ against ALL reflections. The weighted *R*-factor *wR* and goodness of fit *S* are based on *F*^2^, conventional *R*-factors *R* are based on *F*, with *F* set to zero for negative *F*^2^. The threshold expression of *F*^2^ > σ(*F*^2^) is used only for calculating *R*-factors(gt) *etc*. and is not relevant to the choice of reflections for refinement. *R*-factors based on *F*^2^ are statistically about twice as large as those based on *F*, and *R*- factors based on ALL data will be even larger.

### Fractional atomic coordinates and isotropic or equivalent isotropic displacement parameters (Å^2^)

**Table:**

	*x*	*y*	*z*	*U*_iso_*/*U*_eq_	
S1	0.37723 (6)	0.43126 (6)	0.61955 (4)	0.0582 (3)	
N3	0.39660 (16)	0.21826 (17)	0.81129 (9)	0.0356 (4)	
N1	0.61089 (18)	0.2561 (2)	0.78623 (10)	0.0442 (5)	
N4	0.01247 (18)	−0.0268 (2)	0.74184 (11)	0.0456 (5)	
N2	0.60666 (18)	0.29618 (19)	0.86153 (10)	0.0437 (5)	
C4	0.3386 (2)	0.2567 (2)	0.62899 (11)	0.0410 (5)	
C7	0.4785 (2)	0.2729 (2)	0.87518 (11)	0.0393 (5)	
N5	−0.1397 (2)	0.0259 (3)	0.63795 (14)	0.0662 (6)	
C9	0.2487 (2)	0.1719 (2)	0.80399 (12)	0.0410 (5)	
H9A	0.2041	0.1797	0.7507	0.049*	
H9B	0.1983	0.2336	0.8342	0.049*	
C5	0.4407 (2)	0.1592 (2)	0.67757 (12)	0.0465 (5)	
H5A	0.3968	0.0672	0.6789	0.056*	
H5B	0.5246	0.1482	0.6537	0.056*	
C3	0.2087 (2)	0.2246 (3)	0.58660 (12)	0.0494 (6)	
H3	0.1678	0.1355	0.5844	0.059*	
C12	0.0596 (2)	−0.0922 (3)	0.68159 (14)	0.0501 (6)	
H12	0.1401	−0.1480	0.6836	0.060*	
C8	0.4268 (3)	0.3028 (3)	0.94823 (13)	0.0592 (7)	
H8A	0.5039	0.3365	0.9855	0.089*	
H8B	0.3892	0.2180	0.9668	0.089*	
H8C	0.3538	0.3731	0.9397	0.089*	
C10	0.2361 (2)	0.0211 (2)	0.83060 (12)	0.0446 (5)	
H10A	0.2792	0.0138	0.8842	0.053*	
H10B	0.2880	−0.0403	0.8011	0.053*	
C6	0.4841 (2)	0.2100 (2)	0.75747 (11)	0.0372 (5)	
C2	0.1441 (2)	0.3447 (3)	0.54633 (14)	0.0570 (6)	
H2	0.0564	0.3420	0.5145	0.068*	
C1	0.2227 (3)	0.4610 (3)	0.55909 (15)	0.0586 (6)	
H1	0.1960	0.5483	0.5374	0.070*	
C13	−0.0351 (3)	−0.0591 (3)	0.61877 (15)	0.0588 (7)	
H13	−0.0299	−0.0897	0.5692	0.071*	
C11	0.0822 (2)	−0.0285 (3)	0.82172 (13)	0.0548 (6)	
H11A	0.0798	−0.1236	0.8417	0.066*	
H11B	0.0305	0.0320	0.8518	0.066*	
C14	−0.1074 (2)	0.0419 (3)	0.71219 (17)	0.0590 (7)	
H14	−0.1611	0.0948	0.7413	0.071*	
O1W	0.8146 (3)	0.2886 (3)	1.00449 (18)	0.1142 (11)	
H1W	0.758 (3)	0.312 (4)	0.9581 (11)	0.111 (13)*	
H2W	0.801 (5)	0.341 (5)	1.0473 (14)	0.16 (2)*	

### Atomic displacement parameters (Å^2^)

**Table:**

	*U*^11^	*U*^22^	*U*^33^	*U*^12^	*U*^13^	*U*^23^
S1	0.0574 (4)	0.0422 (4)	0.0688 (5)	−0.0088 (2)	−0.0084 (3)	−0.0012 (3)
N3	0.0323 (8)	0.0379 (9)	0.0360 (8)	0.0000 (7)	0.0033 (6)	0.0008 (7)
N1	0.0393 (9)	0.0478 (11)	0.0460 (10)	−0.0052 (8)	0.0085 (7)	0.0062 (8)
N4	0.0333 (9)	0.0503 (11)	0.0529 (11)	−0.0075 (8)	0.0058 (7)	0.0015 (8)
N2	0.0399 (9)	0.0454 (10)	0.0439 (9)	−0.0064 (8)	0.0011 (7)	0.0028 (8)
C4	0.0495 (11)	0.0409 (11)	0.0327 (10)	−0.0069 (9)	0.0067 (8)	−0.0032 (8)
C7	0.0404 (10)	0.0398 (11)	0.0361 (10)	−0.0002 (8)	0.0017 (8)	0.0022 (8)
N5	0.0560 (13)	0.0636 (14)	0.0717 (14)	−0.0045 (11)	−0.0115 (11)	0.0065 (11)
C9	0.0293 (10)	0.0478 (12)	0.0450 (11)	0.0017 (8)	0.0031 (8)	0.0009 (9)
C5	0.0562 (13)	0.0422 (12)	0.0415 (11)	0.0023 (10)	0.0093 (10)	−0.0024 (9)
C3	0.0529 (13)	0.0553 (14)	0.0406 (11)	−0.0170 (10)	0.0090 (9)	−0.0014 (10)
C12	0.0404 (11)	0.0507 (13)	0.0604 (14)	−0.0112 (10)	0.0117 (10)	−0.0003 (11)
C8	0.0567 (14)	0.0789 (18)	0.0412 (12)	−0.0017 (13)	0.0053 (10)	−0.0093 (11)
C10	0.0368 (11)	0.0516 (13)	0.0440 (11)	−0.0037 (9)	0.0025 (8)	0.0046 (9)
C6	0.0394 (10)	0.0334 (10)	0.0393 (10)	0.0016 (8)	0.0081 (8)	0.0050 (8)
C2	0.0408 (12)	0.0800 (18)	0.0484 (13)	−0.0049 (11)	0.0021 (10)	−0.0044 (12)
C1	0.0578 (14)	0.0577 (15)	0.0579 (14)	0.0098 (12)	0.0017 (11)	0.0059 (11)
C13	0.0603 (15)	0.0606 (15)	0.0547 (14)	−0.0212 (12)	0.0066 (11)	−0.0003 (11)
C11	0.0428 (12)	0.0710 (16)	0.0514 (13)	−0.0128 (11)	0.0103 (10)	0.0055 (11)
C14	0.0404 (12)	0.0542 (15)	0.0798 (18)	−0.0017 (10)	0.0017 (11)	−0.0041 (12)
O1W	0.1007 (18)	0.106 (2)	0.113 (2)	0.0367 (15)	−0.0494 (16)	−0.0440 (17)

### Geometric parameters (Å, °)

**Table:**

S1—C1	1.699 (3)	C5—H5B	0.9700
S1—C4	1.711 (2)	C3—C2	1.429 (4)
N3—C7	1.365 (3)	C3—H3	0.9300
N3—C6	1.366 (3)	C12—C13	1.349 (4)
N3—C9	1.466 (2)	C12—H12	0.9300
N1—C6	1.311 (3)	C8—H8A	0.9600
N1—N2	1.389 (3)	C8—H8B	0.9600
N4—C14	1.348 (3)	C8—H8C	0.9600
N4—C12	1.369 (3)	C10—C11	1.528 (3)
N4—C11	1.458 (3)	C10—H10A	0.9700
N2—C7	1.306 (3)	C10—H10B	0.9700
C4—C3	1.376 (3)	C2—C1	1.333 (4)
C4—C5	1.507 (3)	C2—H2	0.9300
C7—C8	1.481 (3)	C1—H1	0.9300
N5—C14	1.305 (4)	C13—H13	0.9300
N5—C13	1.369 (4)	C11—H11A	0.9700
C9—C10	1.516 (3)	C11—H11B	0.9700
C9—H9A	0.9700	C14—H14	0.9300
C9—H9B	0.9700	O1W—H1W	0.933 (10)
C5—C6	1.484 (3)	O1W—H2W	0.931 (10)
C5—H5A	0.9700		
			
C1—S1—C4	92.43 (12)	C7—C8—H8A	109.5
C7—N3—C6	105.21 (16)	C7—C8—H8B	109.5
C7—N3—C9	126.96 (17)	H8A—C8—H8B	109.5
C6—N3—C9	127.76 (17)	C7—C8—H8C	109.5
C6—N1—N2	107.03 (16)	H8A—C8—H8C	109.5
C14—N4—C12	106.5 (2)	H8B—C8—H8C	109.5
C14—N4—C11	127.4 (2)	C9—C10—C11	112.50 (19)
C12—N4—C11	126.1 (2)	C9—C10—H10A	109.1
C7—N2—N1	107.71 (16)	C11—C10—H10A	109.1
C3—C4—C5	128.1 (2)	C9—C10—H10B	109.1
C3—C4—S1	110.58 (17)	C11—C10—H10B	109.1
C5—C4—S1	121.31 (16)	H10A—C10—H10B	107.8
N2—C7—N3	109.94 (18)	N1—C6—N3	110.11 (17)
N2—C7—C8	125.73 (19)	N1—C6—C5	125.08 (18)
N3—C7—C8	124.32 (19)	N3—C6—C5	124.80 (18)
C14—N5—C13	104.7 (2)	C1—C2—C3	112.9 (2)
N3—C9—C10	112.32 (16)	C1—C2—H2	123.5
N3—C9—H9A	109.1	C3—C2—H2	123.5
C10—C9—H9A	109.1	C2—C1—S1	112.2 (2)
N3—C9—H9B	109.1	C2—C1—H1	123.9
C10—C9—H9B	109.1	S1—C1—H1	123.9
H9A—C9—H9B	107.9	C12—C13—N5	110.7 (2)
C6—C5—C4	113.35 (18)	C12—C13—H13	124.7
C6—C5—H5A	108.9	N5—C13—H13	124.7
C4—C5—H5A	108.9	N4—C11—C10	112.13 (18)
C6—C5—H5B	108.9	N4—C11—H11A	109.2
C4—C5—H5B	108.9	C10—C11—H11A	109.2
H5A—C5—H5B	107.7	N4—C11—H11B	109.2
C4—C3—C2	111.9 (2)	C10—C11—H11B	109.2
C4—C3—H3	124.1	H11A—C11—H11B	107.9
C2—C3—H3	124.1	N5—C14—N4	112.3 (2)
C13—C12—N4	105.7 (2)	N5—C14—H14	123.8
C13—C12—H12	127.1	N4—C14—H14	123.8
N4—C12—H12	127.1	H1W—O1W—H2W	116.6 (17)
			
C6—N1—N2—C7	0.0 (2)	C4—C5—C6—N3	68.3 (3)
C1—S1—C4—C3	−0.50 (17)	C4—C3—C2—C1	−0.6 (3)
C1—S1—C4—C5	178.72 (18)	C3—C2—C1—S1	0.2 (3)
N1—N2—C7—N3	0.0 (2)	C4—S1—C1—C2	0.2 (2)
N1—N2—C7—C8	−179.0 (2)	N4—C12—C13—N5	0.3 (3)
C6—N3—C7—N2	−0.1 (2)	C14—N5—C13—C12	−0.5 (3)
C9—N3—C7—N2	177.05 (18)	C14—N4—C11—C10	−123.4 (3)
C6—N3—C7—C8	179.0 (2)	C12—N4—C11—C10	54.5 (3)
C9—N3—C7—C8	−3.9 (3)	C9—C10—C11—N4	61.6 (3)
C7—N3—C9—C10	−85.2 (2)	C13—N5—C14—N4	0.5 (3)
C6—N3—C9—C10	91.3 (2)	C12—N4—C14—N5	−0.3 (3)
C3—C4—C5—C6	−126.8 (2)	C11—N4—C14—N5	177.9 (2)
S1—C4—C5—C6	54.2 (2)	C3—C4—C5—C6	−126.8 (2)
C5—C4—C3—C2	−178.5 (2)	S1—C4—C5—C6	54.2 (2)
S1—C4—C3—C2	0.7 (2)	C4—C5—C6—N3	68.3 (3)
C14—N4—C12—C13	0.0 (2)	C4—C5—C6—N1	−110.2 (2)
C11—N4—C12—C13	−178.3 (2)	C5—C6—N3—C9	4.3 (3)
N3—C9—C10—C11	−178.95 (18)	C6—N3—C9—C10	91.3 (2)
N2—N1—C6—N3	0.0 (2)	N3—C9—C10—C11	−178.95 (18)
N2—N1—C6—C5	178.65 (19)	C9—C10—C11—N4	61.6 (3)
C7—N3—C6—N1	0.0 (2)	C10—C11—N4—C12	54.5 (3)
C9—N3—C6—N1	−177.03 (18)	C10—C11—N4—C14	−123.4 (3)
C7—N3—C6—C5	−178.63 (19)	C8—C7—N3—C9	−3.9 (3)
C9—N3—C6—C5	4.3 (3)	C7—N3—C9—C10	−85.2 (2)
C4—C5—C6—N1	−110.2 (2)		

### Hydrogen-bond geometry (Å, °)

**Table:**

*D*—H···*A*	*D*—H	H···*A*	*D*···*A*	*D*—H···*A*
O1W—H2W···N5^i^	0.93 (1)	2.04 (2)	2.915 (4)	155 (4)
O1W—H1W···N2	0.93 (1)	2.05 (2)	2.948 (3)	161 (4)

Symmetry codes: (i) *x*+1, −*y*+1/2, *z*+1/2.
